# Down-regulation of C35 decreased the cell viability and migration of breast ductal carcinoma cells

**DOI:** 10.1371/journal.pone.0183941

**Published:** 2017-08-31

**Authors:** Wen Che, Yun Bao, Feng Tang

**Affiliations:** Huashan Hospital of Fudan University, Shanghai, China; State University of New York, UNITED STATES

## Abstract

**Background:**

Resistance to human epidermal growth factor receptor-2 (HER2)-targeted therapies is common, and results in treatment failure and new tumor progression. C35 is over-expressed in many invasive breast cancer endogenously, and functions as an oncogene in breast cancer cell lines.

**Methods:**

The current study aims to investigate effects of C35 on cell viability and migration of HER2-positive breast tumor cells and explore possible mechanisms. The protein expression of C35 in BT-474 breast ductal carcinoma cells was interfered by siRNAs. MTT assay was utilized to detect the viability of BT474 breast ductal carcinoma cells after transfection. The migration of BT474 breast ductal carcinoma cells was examined by cell wound scratch assay.

**Results:**

In addition, the protein expression of C35, HER2, AKT and p-AKT in breast ductal carcinoma cells were detected by Western Blot. The protein expression of C35 was weaker in siRNA1 and siRNA2 groups compared with the control group. The relative expression ratio of C35 to β-actin decreased significantly in siRNA2 and siRNA1 groups. When C35 expression was interfered by siRNA1 and siRNA2 for 48 h, the viability of BT474 breast ductal carcinoma cells decreased compared with the control group. In addition, the migration of breast ductal carcinoma cells decreased when C35 expression was interfered by siRNA1 for 24 h, and interfered by siRNA1 and siRNA2 for 48 h.

## Introduction

Breast cancer is the most common invasive cancer in women, and it affects about 12% of women worldwide [1]. Untreated, human epidermal growth factor receptor-2 (HER2)-positive breast cancers are generally more aggressive than HER2-negative breast cancers [2]. HER2-targeted therapies have improved the prognosis of HER2-positive breast cancer over the last few years [3]. However, resistance to the therapies is common, which results in treatment failure and new tumor progression [4]. Therefore, exploring new therapeutic approaches to treat HER-positive breast cancers is highly needed.

C35 is a 12 kDa membrane-anchored protein that is newly discovered. It is located on the HER2 amplicon between HER2 and GRB7, and functions as an oncogene in breast cancer cell lines [5]. High levels of C35 mRNA expression in primary breast cancer are associated with HER2 gene amplification, and hallmarks of transformation, including invasion into collagen matrix, colony growth in soft agar, and formation of large acinar structures in three-dimensional cell cultures. In addition, the transformed phenotype is associated with epithelial to mesenchymal transition and down-regulation of epithelial markers, including keratin-8 and E-cadherin [5, 6]. Recent studies have shown that the viability of breast carcinoma cells could be affected by various agents, such as factors secreted by osteo-differentiating mesenchymal stem cells [7], oxidized gamma-tocotrienol [8], marine-derived fungus clonostachys rosea [9], inhibition of glucose transporter 4 (GLUT4) [10], fucoxanthin and fucoxanthinol [11], and knockdown of human RNA helicase DDX3 [12]. However, it remains unknown about the effects of C35 down-regulation on the viability and migration of HER2-positive breast tumor cells. To establish C35 as a biomarker and therapeutic target for breast cancer treatment, it is important to explore the functions of the novel protein.

HER2, Akt, and phosphorylated-Akt (p-Akt) regulate the proliferation and migration of breast cancer cells. HER2 is a member of the human epidermal growth factor receptor family. Amplification or over-expression of HER2 plays an important role in the development and progression of aggressive types of breast cancer. HER2 is an important biomarker and target of therapy for about 30% of breast cancer patients [13]. In addition, Akt, also known as protein kinase B (PKB), is a serine/threonine-specific protein kinase that is essential in cellular processes including cell proliferation and migration, apoptosis, glucose metabolism, and transcription. Akt regulates cellular metabolism and survival by binding and regulating various downstream effectors, such as nuclear factor-kappa B (NF-κB), Bcl-2 family proteins, and murine double minute 2 (MDM2) [14]. Akt promotes growth factor-mediated cell survival. Akt phosphorylates BAD, a pro-apoptotic protein of the Bcl-2 family, on Ser136, and it makes BAD dissociate from the Bcl-2/Bcl-X complex and lose the pro-apoptotic function [15]. Akt also activates NF-κB by regulating IκB kinase (IKK), resulting in transcription of pro-survival genes [16].

Therefore, the current study aims to investigate effects of C35 down-regulation on cell viability and migration, and protein expression of HER2, AKT and p-AKT in HER2-positive breast tumor cells.

## Methods

### Cells and reagents

Cells: BT-474 human breast ductal carcinoma cells (Chinese Academy of Science, Shanghai, China). BT-474 cells were cultured with 5% CO_2_ at 37°C in culture medium that is composed of 90% of RPMI-1640 and 10% of fetal bovine serum (FBS), supplemented by 1.5 g/L of NaHCO_3_, 2.5 g/L of glucose, and 0.11 g/L of sodium pyruvate. Cells were digested by 0.25% pancreatic protease, and observed under light microscope.

Reagents: RPMI-1640 culture medium (Gibco Inc., Billings, MT, USA); fetal bovine serum (FBS; Gibco Inc., Billings, MT, USA); MTT powder (Aladdin Inc., Shanghai, China); Lipofectamine 2000 Reagent (Gibco Inc., Billings, MT, USA); RIPA tissue lysis buffer (Beyotime Inc., Shanghai, China); primary antibody of β-actin (ZSGB-Bio Inc., Beijing, China); primary antibody of C35 (Abcam Inc., Cambridge, MA, USA); primary antibody of HER2 (Cell Signaling Technology Inc., Danvers, MA, USA); primary antibody of Akt (Cell Signaling Technology Inc., Danvers, MA, USA); primary antibody of p-Akt (Cell Signaling Technology Inc., Danvers, MA, USA); goat anti-mice secondary antibody (Santa Cruz Inc., Dallas, TX, USA); goat anti-rabbit secondary antibody (Santa Cruz Inc., Dallas, TX, USA). C35 siRNA (GenePharma Inc., Shanghai, China) was dissolved in 62.5 μL DEPC to make the final concentration of 20 μM, and the final solution was stored at -20°C.

Equipments: freeze centrifuge (USTC ZONKIA Inc., Hefei, China); cell culture incubator (Thermo Electron Inc., Waltham, MA, USA); thermostatic drying oven (Huyue Inc., Shangyu, China); light microscope (Olympus Inc., Tokyo, Japan); microplate reader (Kehua Inc., Shanghai, China); electrophoresis apparatus (Liuyi Inc., Beijing, China); transfer apparatus (Liuyi Inc., Beijing, China); ice-maker (Xuehua Inc., Changshu, China).

### siRNA transfection

C35 siRNA sequences were synthesized by GenePharma Inc. (Shanghai, China). C35 siRNA1: (Sense: 5’-CUAGCUCAUUGAGGCCAUCTT-3’; Antisense: 5’-GAUGGCCUCAAUGAGCUAGTT-3’); C35 siRNA2: (Sense: 5’-GAGCCAGUAAUGGAGAAACTT-3’; Antisense: 5’-GUUUCUCCAUUACUGGCUCTT-3’). BT474 cells were cultured in 6-well plates. After culturing for 24 h, the original medium was changed into medium without serum or antibiotics. Transfection reagents were mixed with siRNA. For each well, 10 μL of Lipofectamine 2000 were diluted with 250μL of Opti-MEM, and incubated at room temperature for 5 min. And 10 μL of siRNA were diluted with 250μL of Opti-MEM, and mixed gently. The diluted Lipofectamine 2000 and diluted siRNA were mixed and incubated at room temperature for 20 min. Cells were divided into 5 groups: (1) nothing was added; (2) Lipofectamine 2000 was added; (3) Lipofectamine 2000 and negative control siRNA were added; (4) Lipofectamine 2000 and C35 siRNA1 were added; (5) Lipofectamine 2000 and C35 siRNA2 were added. After transfection for 6 h, the culture medium was changed back to medium with antibiotics. After 48 h of transfection, cells were scratched gently into 1.5 mL Eppendorf tubes, and centrifuged at 12,000 r / min for 5 min. Cell precipitates were stored at -20°C.

### MTT assay

MTT assay was used to detect the viability of BT474 cells after transfection. Cells were placed in 96-well plates. After 24 h of transfection, 20 μL of MTT (5 mg/mL) was added into each well. Four hours later, cell culture medium was discarded, and 150 μL of DMSO was added. Absorbance was detected at 570 nm (A570) using ELISA microplate reader. The relative cell viability was calculated using the following formula: relative cell viability (%) = absorbance of experimental groups / absorbance of the control group×100%.

### Cell wound scratch assay

Three horizontal lines were drawn at the back of 6-well plates using marker pens, and 200 μL tips were utilized to draw horizontal lines at the bottom of plates. The plates were rinsed with phosphate buffer solution (PBS) for 3 times to eliminate cells peeled off during drawing. Sample and take photos at 0, 24, and 48 h. The distance of migration and migration index was calculated using Image-Pro Plus 6.0 software (Media Cybernetics Inc., Rockville, MD). Calculation formula: the inhibition rate of cell migration (%) = (1 –migration distance of experimental groups / migration distance of the control group) ×100%.

### Western blot

Protein expression of C35, HER2, AKT, and p-AKT were detected by Western Blot. Cells were washed with phosphate-buffered solution (PBS), and scratched into 1.5 mL Eppendorf tubes. Cells were then centrifuged at 12, 000 rpm, 4°C for 5 min. Cell pellet was washed and lysis buffer with protease and phosphatase was added at 4°C. The mixture was then centrifuged at 12, 000 rpm, 4°C for 10 min. The protein concentration in the supernatant was detected, and the supernatant was then stored in a-80°C fridge. Polyacrylamide gel electrophoresis (PAGE) was made according to different molecular weight of proteins. High-concentration PAGE was used for proteins with low molecular weight, whereas low-concentration PAGE was utilized for proteins with high molecular weight. Samples were placed in boiling water for 10 min, and supernatant was loaded. Twenty μg of proteins was loaded into each well. Proteins were then separated at 120 V for 20 min in condensing gel, and at 180 V for 60 min in separating gel. The transfer buffer was made of 25 mM of Tris, 192 mM of glycine, 20% of ethanol. Signal was transferred to polyvinylidene fluoride (PVDF) membranes at 100 V for 120 min. After being blocked with 5% fat free milk powder for 1 h, membranes were incubated with primary antibodies for C35, HER2, AKT, and p-AKT at 4°C overnight. Membranes were incubated with goat anti-rabbit secondary antibody (1:4000) with HRP at room temperature for 1 h. Membranes were incubated shortly with electro-chemi-luminescence (ECL) solution, and films were exposed in a dark room.

### Quantitative polymerase chain reaction (qPCR)

Total RNA of BT-474 cells was extracted and purified by Trizol according to manufacturer's instructions. cDNA synthesis kit (Tiangene Biotech Inc., Beijing, China) was used for reverse transcription. Each reaction contained 1 μL of random hexamer primer (0.2 μg/μL) and 40 U M-MuLV reverse transcriptase (20 U/μL). The specific primer for detection of HER2 gene was F: AGAAGCCCTGATGTGTCCTC; R: GTGCTGTTCCTCTTCCAACG. The primer for AKT gene was F: GGACAACCGCCATCCAGACTG; R: TTGGCCAGGGACACCTCCATC. The primer for β-actin gene was F: AGAAAATCTGGCACCACACC; R: AGAGGGTACAGGGATAGCA. MiRcute miRNA qPCR Detection kit was utilized for qPCR. PCR conditions were: pre-denaturing at 95°C for 15 min; denaturing at 95°C for 10 s; and annealing and polymerization at 60 to 66°C for 20 to 32 s. There were 40 PCR cycles. PCR was performed in an ABI Step One Plus qPCR System. The expression of HER2 and AKT was determined by the ratio of relative optical density of target genes to β-actin.

### Construction of C35 overexpression vector

Primer STAR HS DNA polymerase (Takara Bio Inc., Shiga, Japan) was utilized in PCR. 5’ of the gene had XhoI enzyme cutting site, and 3’ had HindIII enzyme cutting site. The primer was F: CCGCTCGAGATGAGCGGGGAGCCGGGG; R: CCCAAGCTTTCACAGGATGACGCAGGG. 1436 pcDNA3 Flag HA vector was purchased from Addgene Inc. (Cambridge, MA, USA). XhoI and HindIII were used for dual-enzyme digestion, and agarose gel extraction kit was utilized for gathering DNA. The concentration of DNA was detected, and mixed with target genes. Restriction enzyme alone served for the control group. The reaction was carried out at 16°C for 1 h. The products in two groups were transformed into DH5α cells at 37°C overnight. Monoclonal cells were selected, and cultured in medium at 37°C and 200 r/min for 8 to 16 h. Plasmids in the culture medium were extracted. XhoI and HindIII were utilized to digest products, and agarose electrophoresis was used to examine whether target genes were inserted or not. BT-474 cells that were interfered by siRNA1 were then transfected with vectors with C35 overexpression. Cell viability and migration were tested.

### Statistical analysis

Results were demonstrated as mean ± SEM. One way analysis of variance (ANOVA) was used to compare differences among three or more groups, followed by Bonferroni post hoc testing for multiple comparisons. *p* values of 0.05 or less were regarded as significant. Statistical analysis and figures were made by GraphPad Prism 5.0 software (GraphPad Software Inc., La Jolla, CA).

## Results

### The efficacy of interfering C35 expression

**Fig 1:**
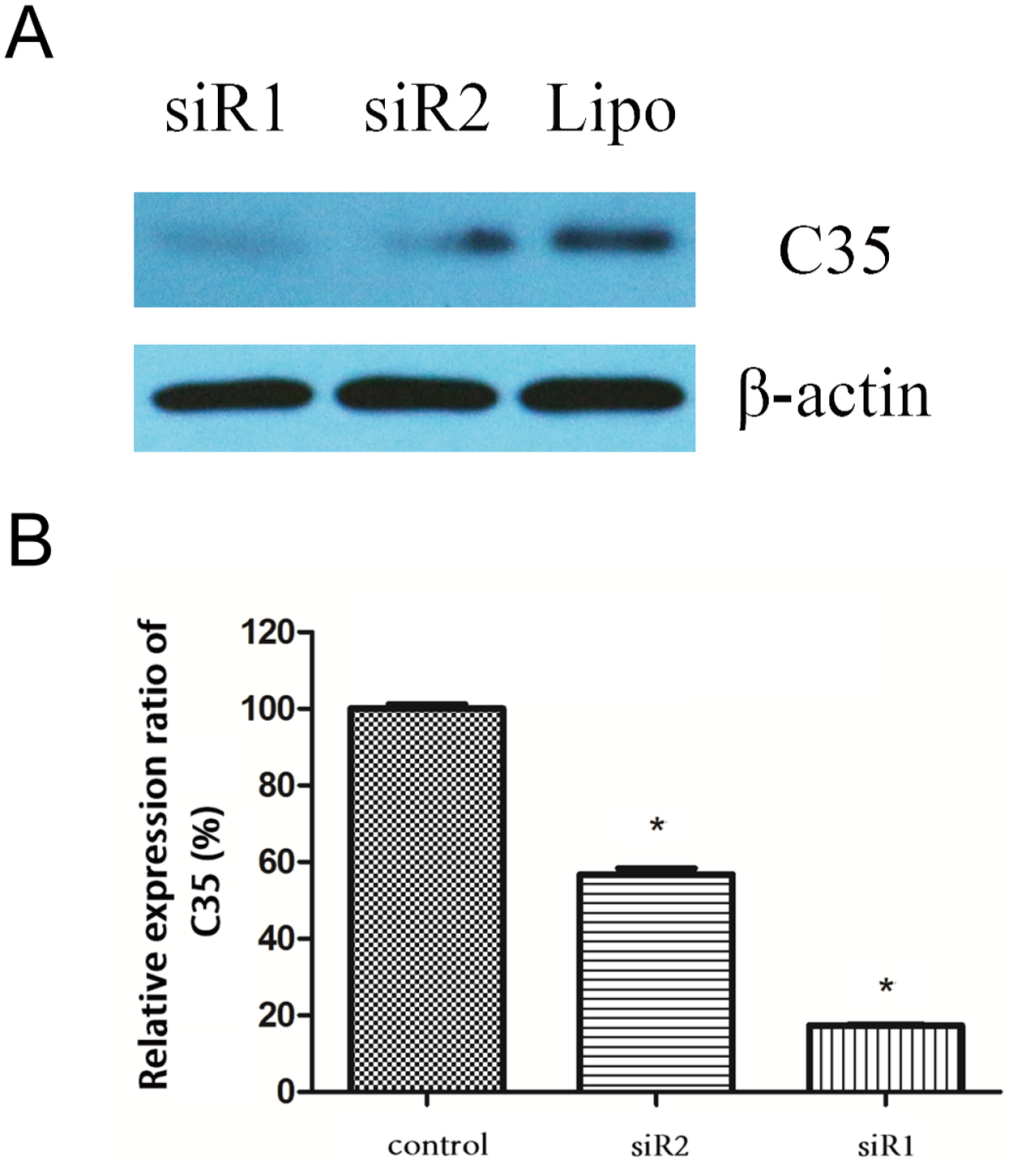
The protein expression of C35 in breast ductal carcinoma cells after siRNA treatment. **(A)** The protein expression of C35 in BT-474 breast ductal carcinoma cells was detected by Western Blot. The protein expression of C35 was weaker in siRNA1 and siRNA2 groups compared with the control group. siR1: siRNA1; siR2: siRNA2. **(B)**The relative expression ratio of C35 to β-actin decreased significantly in siRNA2 and siRNA1 groups. * indicates that *p* < 0.05 as compared with the control group. siR1: siRNA1; siR2: siRNA2.

The protein expression of C35 in BT-474 breast ductal carcinoma cells was detected by Western Blot. Results showed that the protein expression of C35 was weaker in siRNA1 and siRNA2 groups compared with the control group, indicating that both siRNA1 and siRNA2 interfered the protein expression of C35 (Fig 1A). The relative expression ratio of C35 to β-actin in siRNA2 and siRNA1 groups decreased significantly compared with the control group (56.7±1.6% and 17.4±0.1% versus 100±1.2%; *p* < 0.05, Fig 1B).

### The cell viability and migration of breast ductal carcinoma cells after C35 expression was interfered by siRNA

**Fig 2:**
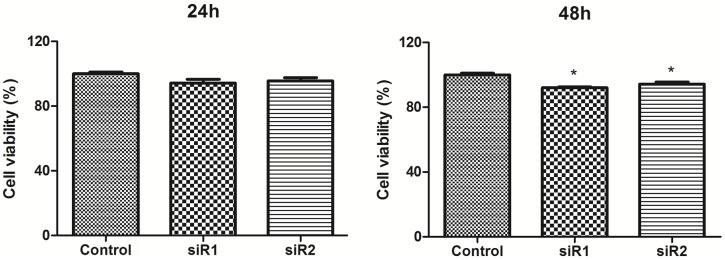
The viability of breast ductal carcinoma cells after C35 expression was interfered by siRNA. MTT assay was used to detect the cell viability of BT474 breast ductal carcinoma cells. When C35 protein was interfered by siRNA1 and siRNA2 for 48 h, the viability of BT474 breast ductal carcinoma cells decreased compared with the control group. * indicates that *p* < 0.05 as compared with the control group. siR1: siRNA1; siR2: siRNA2.

**Fig 3:**
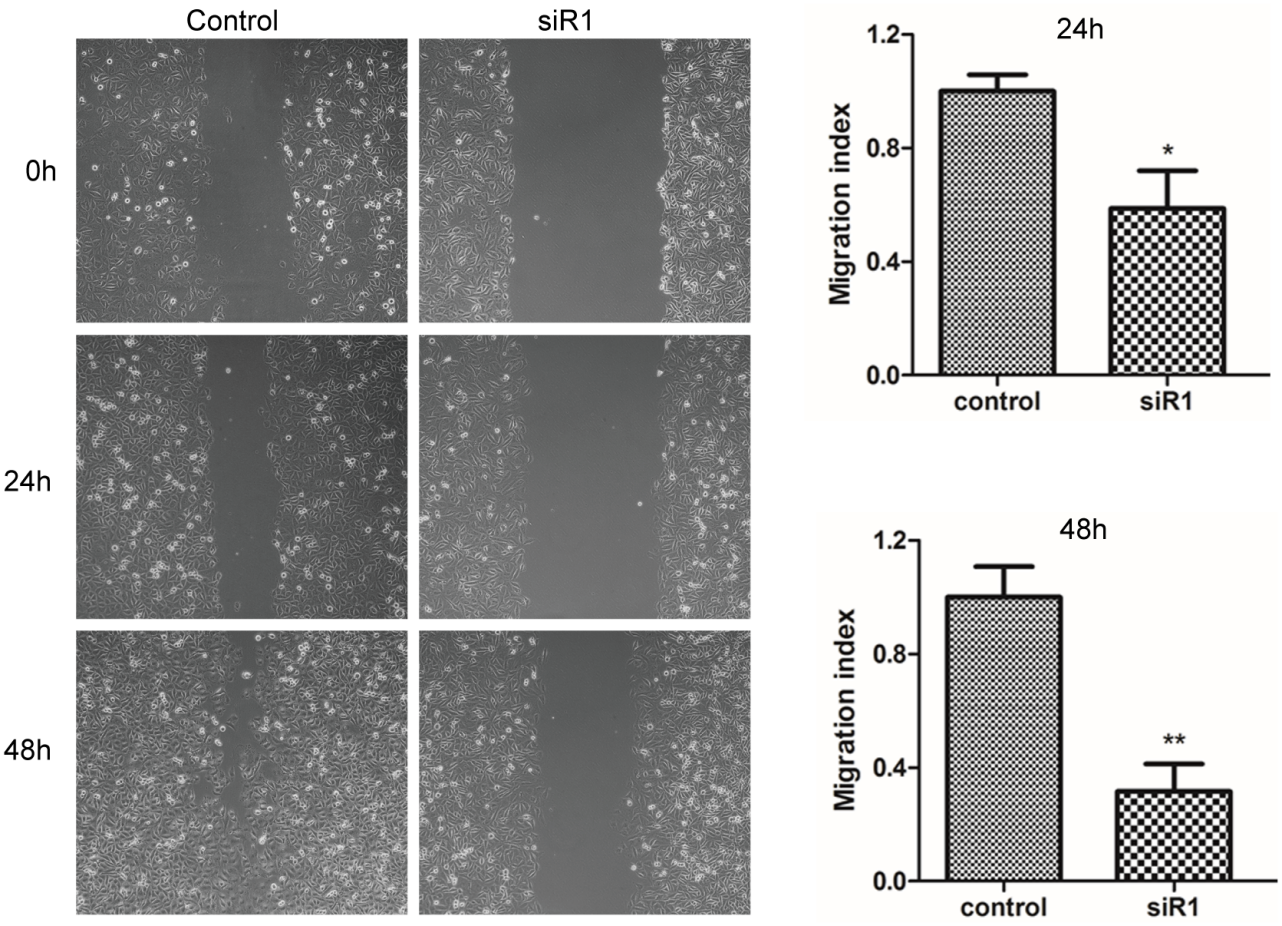
The migration of breast ductal carcinoma cells after C35 expression was interfered by siRNA. Cell wound scratch assay was utilized to examine the migration of BT474 breast ductal carcinoma cells. The migration of breast ductal carcinoma cells decreased when C35 protein was interfered by siRNA1 for 24 h, and for 48 h. * indicates that *p* < 0.05 as compared with the control group. siR1: siRNA1.

MTT assay was used to detect the cell viability of BT474 breast ductal carcinoma cells, and cell wound scratch assay was utilized to examine cell migration. When C35 protein was interfered by siRNA1 and siRNA2 for 48 h or 72h, the viability of BT474 breast ductal carcinoma cells decreased compared with the control group (92.0±0.6% and 94.5±1.3% versus 100±1.2%; *p* < 0.05, Fig 2) or (83.1±3.1% and 88.6±2.3% versus 96.8±0.9%, S1 Fig). The cell viability did not change significantly when C35 protein was interfered for 24 h. In addition, the migration of breast ductal carcinoma cells decreased when C35 protein was interfered by siRNA1 for 24 h (migration index: 0.6±0.1 versus 1.0±0.1; *p* < 0.05, Fig 3), and for 48 h (migration index: 0.3±0.1 versus 1.0±0.1; *p* < 0.05, Fig 3).

### The cell viability and migration of C35 siRNA1-treated breast ductal carcinoma cells after C35 gene was overexpressed

**Fig 4:**
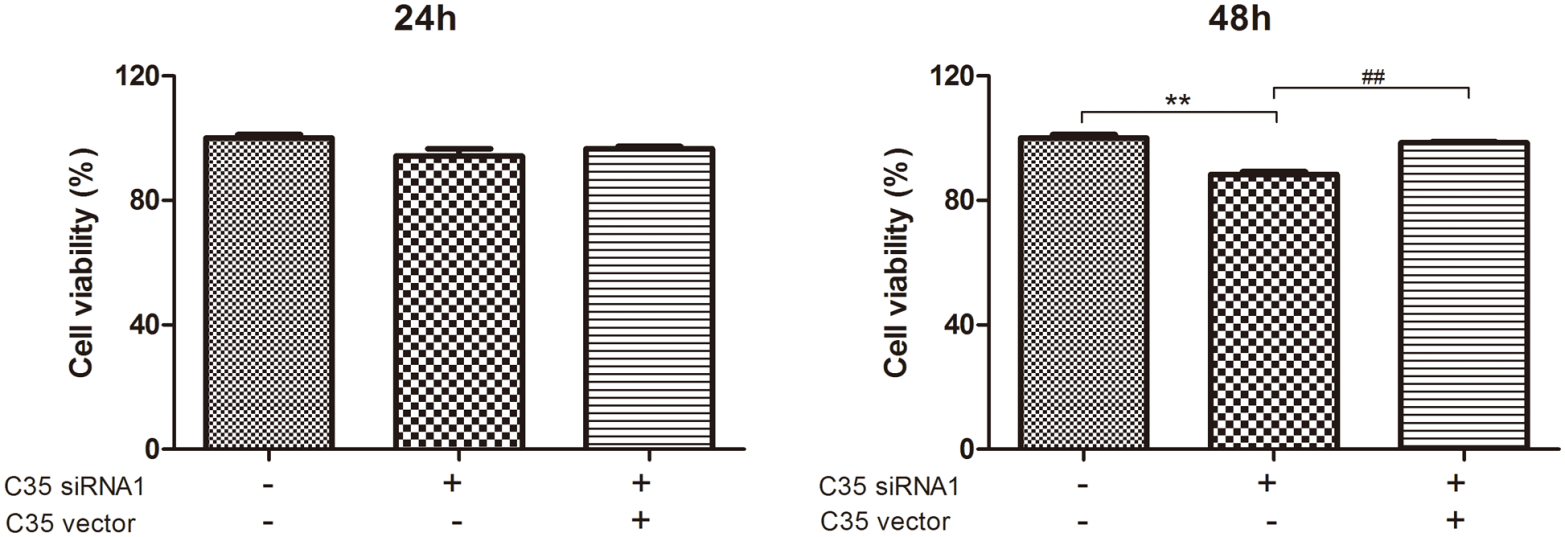
The cell viability of C35 siRNA1-treated breast ductal carcinoma cells after C35 gene was overexpressed. C35 overexpressed vectors were transfected into C35 siRNA1-treated BT-474 cells. The viability of BT-474 cells was detected. The decreased viability in C35 siRNA1-treated BT-474 cells was restored after C35 was overexpressed at 48 h. ** indicates that *p* < 0.01 as compared with the control group. ## indicates that *p* < 0.01 as compared with siRNA1 group alone.

**Fig 5:**
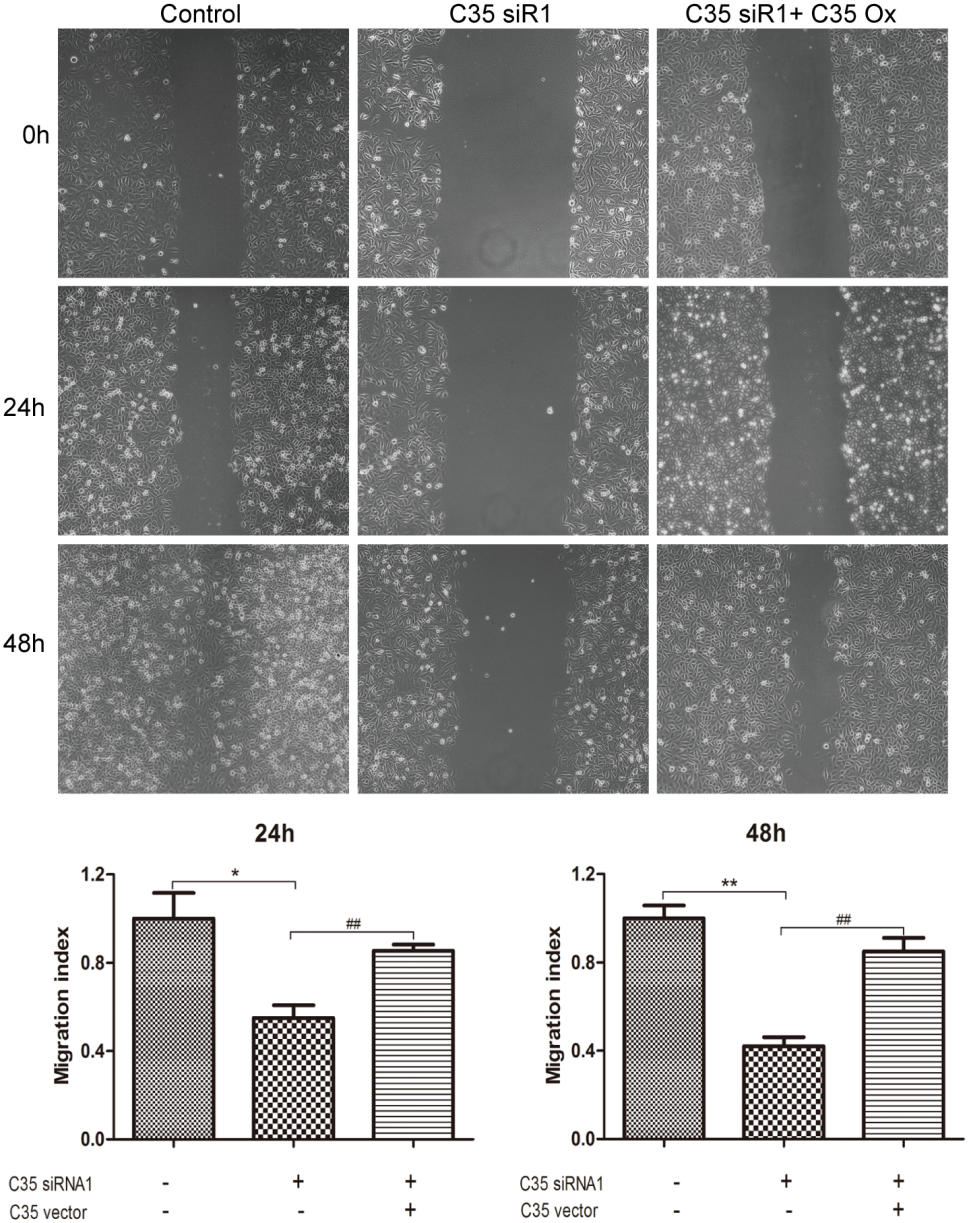
The migration of C35 siRNA1-treated breast ductal carcinoma cells after C35 gene was overexpressed. C35 overexpressed vectors were transfected into C35 siRNA1-treated BT-474 cells. The migration index of BT-474 cells was detected. The decreased migration index in C35 siRNA1-treated BT-474 cells was restored after C35 was overexpressed at 24 and 48 h. * and ** suggest that *p* < 0.05 and *p* < 0.01 respectively as compared with the control group. ## suggests that *p* < 0.01 as compared with siRNA1 alone group. siR1: siRNA1; Ox: Overexpression.

C35 overexpressed vectors were transfected into BT-474 cells with low C35 expression (C35 expression was interfered by siRNA1). The viability and migration of BT-474 cells were detected. The decreased viability in C35 siRNA1-treated BT-474 cells was restored after C35 was overexpressed at 48 h (*p* < 0.01, Fig 4). In addition, the decreased migration index in C35 siRNA1-treated BT-474 cells was restored after C35 was overexpressed at 24 and 48 h (*p* < 0.01, Fig 5). The proteins expression in each group indicate in S2 Fig.

### The protein and mRNA expression of HER2, AKT, and p-AKT decreased after C35 expression was interfered by siRNA

**Fig 6:**
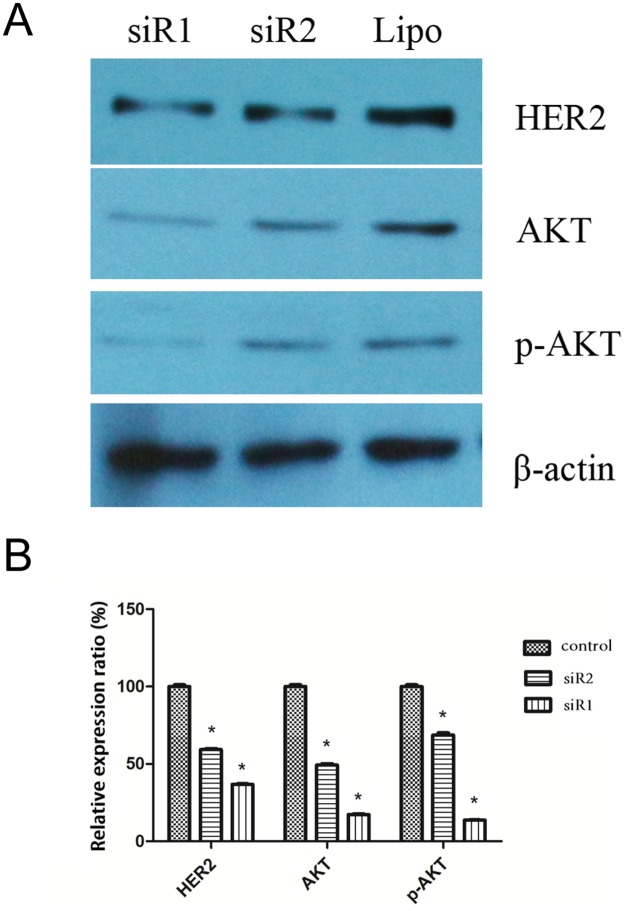
The protein expression of HER2, AKT and p-AKT in breast ductal carcinoma cells after C35 expression was interfered by siRNA. **(A)** Protein expression of HER2, AKT and p-AKT in breast ductal carcinoma cells were detected by Western Blot. The protein expression of HER2, AKT and p-AKT decreased after C35 protein was interfered by siRNA1 and siRNA2. siR1: siRNA1; siR2: siRNA2; HER2: human epidermal growth factor receptor type 2. **(B)** The relative expression ratio of HER2, AKT and p-AKT to β-actin decreased after C35 protein was interfered by siRNA. * indicates that *p* < 0.05 as compared with the control group. siR1: siRNA1; siR2: siRNA2; HER2: human epidermal growth factor receptor type 2.

**Fig 7:**
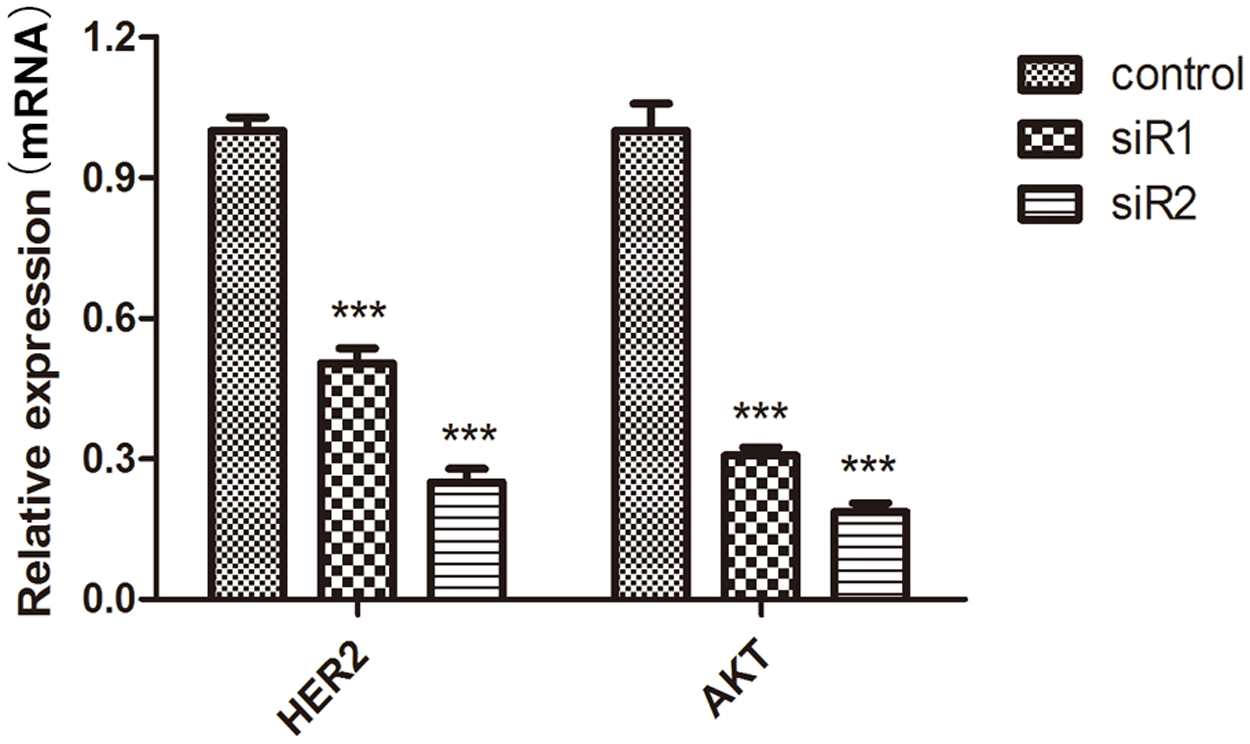
Relative mRNA expression of HER2 and AKT after C35 expression was interfered by siRNA. mRNA levels of HER2 and AKT were examined by qPCR. mRNA levels of HER2 and AKT decreased after expression of C35 was interfered by siRNA1 and siRNA2 as compared with the control group. *** suggests that *p* < 0.001 as compared with the control group. siR1: siRNA1; siR2: siRNA2; HER2: human epidermal growth factor receptor type 2.

Protein expression of HER2, AKT, and p-AKT were detected by Western Blot. mRNA levels of HER2 and AKT were examined by qPCR. The relative protein expression of HER2 (siRNA1: 36.8±0.5%; siRNA2: 59.4±0.7%; control: 100±1.2%), AKT (siRNA1: 17.3±0.5%; siRNA2: 47.9±0.9%; control: 100±1.0%), and p-AKT (siRNA1: 13.7±0.2%; siRNA2: 68.7±1.4%; control: 100±1.2%) to β-actin in breast ductal carcinoma cells decreased after C35 protein was interfered by siRNA1 and siRNA2, compared with the control group (*p* < 0.05, Fig 6A and 6B). Similarly, mRNA levels of HER2 and AKT decreased after expression of C35 was interfered by siRNA1 and siRNA2 as compared with control group (*p* < 0.001, Fig 7). This indicates that C35 up-regulated the protein expression of HER2, AKT and p-AKT, and affected the proliferation and migration of cancer cells in AKT signaling pathway-dependent manner.

## Discussion

We have demonstrated that the cell viability and migration of BT474 breast ductal carcinoma cells and protein expression of HER2, AKT and p-AKT decreased when C35 expression was interfered by siRNA.

C35 is a newly discovered gene, and is located on chromosome 17q12 adjacent to the oncogene that encodes HER2/neu. C35 gene encodes a small 115 amino acid protein with a molecular weight of about 12 kDa, and its functional importance is largely unknown [17]. Immuno-histochemical studies in breast cancer lumpectomy samples demonstrated that the C35 gene was over-expressed in more than 60% of breast cancer tissues, whereas was not apparent in normal tissues. C35 genes were also over-expressed in patients with HER2/neu-negative breast cancer and breast tumor cell lines (5).

To explore the functional importance of C35, siRNAs were utilized in current study to target the protein expression of C35. RNAi has high efficiency and specificity, and is widely used to knockdown target genes in order to examine gene function or explore experimental treatment for diseases [18]. siRNAs could target different sites of the same gene, and their inhibition of gene expression varied. Two siRNAs targeting different regions of the C35 gene were utilized in current study. Results showed that the protein expression of C35 in BT474 breast ductal carcinoma cells was significantly inhibited by these two siRNAs, especially siRNA1.

In current study, we revealed that targeting the protein and mRNA expression of C35 gene could reduce the viability of breast ductal carcinoma cells. This provides a novel therapeutic approach for treating breast cancer. Mechanism study demonstrated that down-regulation of C35 decreased the protein expression of HER2, AKT and p-AKT. The C35 gene locates on chromosome 17q12, and it is 505 nucleotides away from the 3' end of the HER2 oncogene. The chromosomal arrangement of genes encoding C35 and HER2 is tail to tail [5]. Therefore, it is possible that approaches to down-regulate C35 may affect oncogene HER2 as well. Studies have revealed the close relationship between C35 and HER2, which is a key component in regulating cell growth [19, 20]. It was reported that 34% of patients with breast cancer were tested positive for both C35 and HER2, whereas 31% were positive for C35 and negative for HER2. All breast cancer patients who had overexpression of HER2 also had overexpression of C35, whereas C35 was also over-expressed in patients who were negative for HER2/neu [5]. Further experiments are required to elucidate the transcription factors or other components that may have linked the expression of C35 and HER2 together.

In addition, Akt and its active form p-AKT play a key role in cellular survival pathways by inhibiting apoptotic processes. Research about how C35 expression affects Akt pathway is scarce. Given the close location and relationship of C35 and HER2 gene, it is likely that C35 activated oncogene HER2, which then stimulated the pro-survival Akt pathway. Selective inhibition of HER2 was shown to inhibit AKT signal transduction and prolong disease-free survival in a micro-metastasis model of ovarian carcinoma [21]. Meanwhile, the effect of HER2 overexpression on p53 gene expression, cell proliferation and sensitivity to gamma-irradiation was reported to be through PI3K/Akt pathway in breast cancer cell MCF7 [22]. Therefore, the decreased expression of oncogene HER2, and depressed pro-survival Akt pathway after targeting C35 expression may account for the decreased viability of breast carcinoma cells.

In addition, we demonstrated that the migration of BT474 breast ductal carcinoma cells decreased when C35 expression was inhibited. The migration and metastasis of tumor cells correlate with worse prognosis. Our research findings revealed for the first time that the down-regulation of C35 may reduce tumor progression and indicate better prognosis of breast carcinoma. Akt promotes the migration of tumor cells [23], and the reduced expression of Akt and p-Akt after C35 was targeted may result in decreased tumor migration.

## Conclusions

The protein expression of HER2, AKT and p-AKT decreased compared with the control group after C35 expression was interfered by siRNA1 and siRNA2. Down-regulation of C35 decreased the cell viability and migration of BT474 breast ductal carcinoma cells by inhibiting protein expression of HER2, AKT and p-AKT.

## Supporting information

S1 Fig The viability of breast ductal carcinoma cells after C35 expression was interfered by siRNA. MTT assay was used to detect the cell viability of BT474 breast ductal carcinoma cells. When C35 protein was interfered by siRNA1 and siRNA2 for 72 h, the viability of BT474 breast ductal carcinoma cells decreased compared to the control group. * indicates that p < 0.05 as compared to the control group. siR1: siRNA1; siR2: siRNA2.
(TIF)

S2 Fig Western blot detected the protein expression of C35 in BT-474 breast ductal carcinoma cells. (TIF)
