# Supplementary figures and images for: Down-regulation of C35 decreased the cell viability and migration of breast ductal carcinoma cells
Source: PLoS One. 2017 Aug 31;12(8):e0183941. doi: 10.1371/journal.pone.0183941 (PMC5578656; doi:10.1371/journal.pone.0183941)

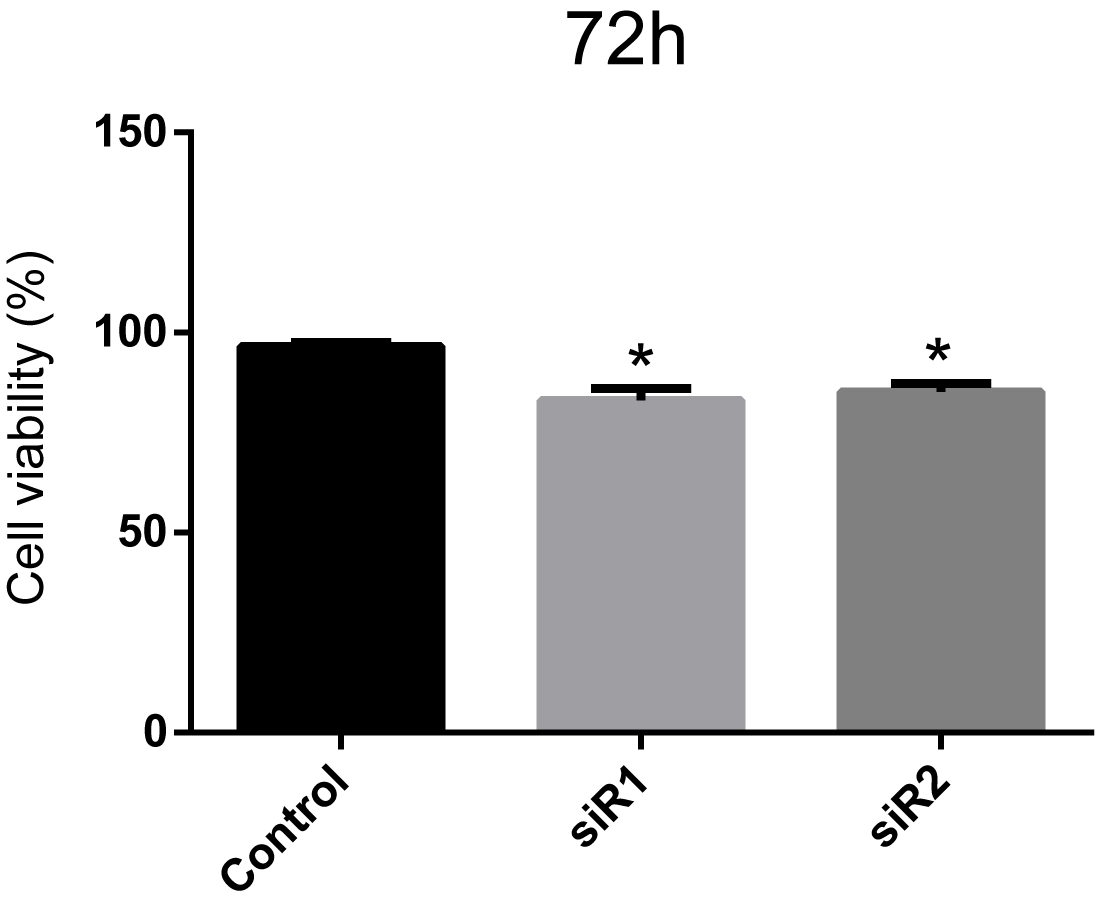

Supplement: S1 Fig — MTT assay was used to detect the cell viability of BT474 breast ductal carcinoma cells. When C35 protein was interfered by siRNA1 and siRNA2 for 72 h, the viability of BT474 breast ductal carcinoma cells decreased compared to the control group. * indicates that p < 0.05 as compared to the control group. siR1: siRNA1; siR2: siRNA2. (TIF) [file pone.0183941.s001.tif]

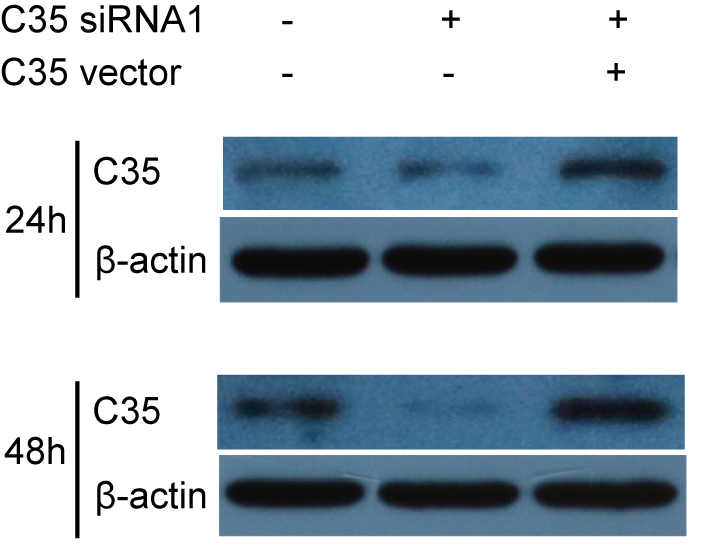

Supplement: S2 Fig — (TIF) [file pone.0183941.s002.tif]
